# Prognostic Models Incorporating *RAS* Mutation to Predict Survival in Patients with Colorectal Liver Metastases: A Narrative Review

**DOI:** 10.3390/cancers14133223

**Published:** 2022-06-30

**Authors:** Geoffrey Yuet Mun Wong, Connie Diakos, Mark P. Molloy, Thomas J. Hugh

**Affiliations:** 1 Department of Upper Gastrointestinal Surgery, Royal North Shore Hospital, Sydney, NSW 2065, Australia; tom.hugh@sydney.edu.au; 2 Northern Clinical School, The University of Sydney, Sydney, NSW 2065, Australia; connie.diakos@sydney.edu.au; 3 Department of Medical Oncology, Royal North Shore Hospital, Sydney, NSW 2065, Australia; 4 Bowel Cancer and Biomarker Research Laboratory, Faculty of Medicine and Health, School of Medical Sciences, The University of Sydney, Sydney, NSW 2006, Australia; m.molloy@sydney.edu.au

**Keywords:** *RAS*, colorectal liver metastases, prognosis, prediction models

## Abstract

Recurrence and survival vary widely among patients who undergo curative-intent resection of colorectal liver metastases (CRLM). Prognostic models provide estimated probabilities of these outcomes and allow the effects of multiple potentially interacting variables to be adjusted and assessed simultaneously. Although many prognostic models based on clinicopathologic factors have been developed since the 1990s to predict survival after resection of CRLM, these models vary in their predictive performance when applied to contemporary cohorts. Rat sarcoma viral oncogene homolog (*RAS*) mutation status is routinely tested in patients with metastatic colorectal cancer to predict response to anti-epidermal growth factor therapy. In addition, mutations in *RAS* predict survival and recurrence in patients undergoing hepatectomy for CRLM. Several recent prognostic models have incorporated *RAS* mutation status as a surrogate of tumor biology and combined revised clinicopathologic variables to improve the prediction of recurrence and survival. This narrative review aims to evaluate the differences between contemporary prognostic models incorporating *RAS* mutation status and their clinical applicability in patients considered for curative-intent resection of CRLM.

## 1. Introduction

The treatment paradigm for patients with colorectal liver metastases (CRLM) depends upon an initial determination of a potentially curative versus palliative approach. The current curative-intent treatment strategy focuses on liver resection in selected patients [1,2]. Both technical and prognostic criteria are considered when evaluating patients for surgery. Technical resectability is the complete resection of intrahepatic and extrahepatic disease while preserving an adequate future liver remnant. The criteria for resectability of CRLM have expanded over time due to advancements in systemic therapy and improvements in resectability using approaches such as portal vein embolization and staged hepatectomy [3,4]. Five- and ten-year survival rates post liver resection for CRLM have been reported at 40 to 58% and 24%, respectively [5,6]. However, even in the setting of a curative-intent liver resection, an estimated 70% of patients develop recurrence, and patient survival varies widely [7,8].

Many prognostic models for predicting survival after hepatic resection for metastatic colorectal cancer (CRC) have been developed since the 1990s [9]. These models simultaneously assess the effects of multiple, potentially interacting clinicopathologic predictors to provide an estimated survival probability. In brief, the key steps in developing these prognostic models are determining the endpoint (e.g., overall survival or recurrence-free survival), preparing the dataset, applying a statistical framework (e.g., multivariable regression), selecting the predictors, coding the predictors, and assessing the relationship between the predictors and outcome [10]. The clinical risk score (CRS) by Fong et al. (1999) is widely regarded as the reference standard and was developed based on an analysis of 1001 consecutive cases undergoing liver resection for CRLM at the Memorial Sloan Kettering Cancer Center between 1985 and 1998. Clinical, pathological, and outcome data from this cohort demonstrated that five preoperative and independent predictors (node-positive primary, the disease-free interval from primary to metastases <12 months, number of hepatic tumors >1, largest hepatic tumor >5 cm, and carcinoembryonic antigen >200) could indeed predict survival accurately and distribute patients along a wide range of survival duration [11]. Although the CRS is widely cited and used for evaluating baseline characteristics of patients in observational studies, advancements in the multimodal treatment of CRLM mean that the patients selected for a curative-intent approach in more recent times differ from cohorts used to develop the CRS. The inconsistent predictive performance of the CRS and risk scores developed from cohorts around the same period reflect a need to revise these models [12,13]. Furthermore, clinicopathologic predictors are imperfect surrogates of tumor biology and lose prognostic value as survival time increases [14].

Newer prognostic models have included revised clinicopathologic predictors and molecular biomarkers in response to these limitations. The Kirsten rat sarcoma viral oncogene homolog (*KRAS*) and neuroblastoma rat sarcoma viral oncogene homolog (*NRAS*) genes are members of the *RAS* gene family and frequently mutated (30% to 40%) in CRC. These *RAS* mutations drive cancer proliferation through epidermal growth factor receptor (EGFR)-independent activation of the mitogen-activated protein kinase (MAPK) signaling pathway [15]. *RAS* testing is routinely tested in tumor specimens as mutation predicts a lack of response to anti-EGFR therapy in patients with metastatic CRC [16,17]. *RAS* mutation is prognostic and is independently associated with worse overall survival and recurrence-free survival among patients who had resection of CRLM [18]. This narrative review assesses the performance of prognostic models incorporating *RAS* mutation status to estimate recurrence and survival after resection of CRLM.

## 2. Prognostic Models Incorporating *RAS* Mutation Status

Using the search engine PubMed and Embase, our strategy to identify relevant literature focused on the following search strings in study titles and keywords: prognostic model, risk score, colorectal cancer, liver metastases, liver resection, *RAS* mutation, and survival. References in the retrieved articles were reviewed to identify articles missed in the electronic search. Information including the first author, publication year, the population in which the prognostic model was developed and validated, predicted outcomes, and the resulting models are summarized in Table 1. The CRS is included for reference as most prognostic models used the CRS to compare the performance of their models [11].

Twelve prognostic models incorporating *RAS* mutation status as a predictor of outcomes after resection of CRLM have been published since 2017 [19,20,21,22,23,24,25,26,27,28,29,30]. Ten models were developed and validated by including patients from 2000 onwards, which parallels key developments in systemic chemotherapy such as the introduction of irinotecan and oxaliplatin as components of cytotoxic combination therapy and the use of biological agents such as bevacizumab, cetuximab, and panitumumab [4]. Although the most recent, largest, and longest longitudinal study to date by Buisman et al., 2020 included patients from a long inclusion period (1992–2019) to estimate 10-year overall survival, the cohort largely reflects contemporary patient selection and treatment as most patients (82.0%) underwent resection after 2000 [30]. Nine studies included a validation cohort [21,22,23,25,26,27,28,29,30]. The predicted outcome was overall survival in eight studies and recurrence-free survival in four studies.

## 3. Assessing the Performance of Prediction Models Incorporating *RAS* Mutations Status

Discrimination and calibration are two key aspects that characterize the performance of a prediction model [31]. These measures were extracted and summarized in Table 2. Discrimination was assessed in 10 studies and calibration was assessed in seven studies. Discrimination refers to how a prediction model distinguishes patients with and without the outcome and is quantified by the concordance (C) statistic or area under the receiver operating characteristic curve (AUC). The C-statistic range from 0.5 to 1. A C-statistic of 0.5 indicates that the prediction model is no better than random chance in distinguishing patients with and without the outcome. At the other end of the spectrum, a C-statistic of one indicates that the prediction model always discriminates against patients who experience the outcome compared to those who do not [32]. The model by Buisman et al. to predict 10-year overall survival documented the highest C-statistic (0.73) in its development cohort, while the remaining nine studies reported a C-statistic between 0.6 and 0.7 [20,21,22,23,25,26,27,28,29,30]. Calibration refers to a model’s accuracy of risk estimates and indicates the extent to which expected and observed outcomes agree [33]. Despite differences in calibration methods, seven studies demonstrated validation curves and reported good calibration of their models [23,25,26,27,29,30,34].

## 4. Molecular Biomarkers and Revised Clinicopathologic Predictors in Prognostic Models for Colorectal Liver Metastases

The *RAS* mutation rate, the hazard ratio of *RAS* mutation, 95.0% confidence intervals, and *RAS* mutational analysis of the included studies are summarized in Table 3. *RAS* mutation rates ranged from 36.3% to 46.2% and are consistent with published studies except for Wang et al., 2017 (63.3%). Wang et al. did not explain the higher *KRAS* mutation rate which may have been influenced by the inclusion of only patients with preoperative chemotherapy and a selection bias of patients with *KRAS* mutation. *RAS* isoforms (*KRAS* and *NRAS*) and codons tested varied between studies, with extended *RAS* profiling (beyond *KRAS* exon 2 and codons 12 and 13) performed in more recent cohorts. The hazard ratio of *RAS* mutation ranged between 1.50 and 2.69.

All prognostic models used the overall *RAS* mutation status as dichotomous variables (wild-type or mutated) and assigned one point for *RAS* mutation. Passot et al., 2017 analyzed 524 patients undergoing curative liver resection with known *RAS* mutation status but only included 212 patients with *RAS* mutation in its multivariable analysis and treatment schema [19]. Three other models had molecular markers and *RAS* mutation [24,26,30]. The e-CS and CERR integrated alterations in *the RAS*-*RAF* signaling pathway (*KRAS*, *NRAS,* and *BRAF*) as a single predictor [24,26]. In contrast, the risk score to predict 10-year overall survival by Buisman et al., 2022 incorporated *KRAS* and BRAF mutational status as two separate predictors of equal weight [30]. The e-CS incorporated alterations in *SMAD* as an additional molecular predictor and is the only prognostic score to include molecular profiling that extends beyond *RAS/*RAF** [24].

Predictors evaluated and included in prognostic models incorporating *RAS* to predict outcomes after resection of CRLM are summarized in Table 4. The independent prognostic factors included in prediction models ranged between three and 15. Independent prognostic factors were not always in multivariable analysis in the final model. For example, positive resection margin was an independent predictor for overall survival in the Genetic and Morphology Evaluation (GAME) score but excluded from the scoring system because this information would not be available preoperatively [21]. Similarly, primary lymph node metastasis was not included in the contour prognostic model because this information is not available when a liver-first approach is used in synchronous CRLM [29]. Tumor morphology (maximum size or number of CRLM or a composite measurement of these variables) was an independent predictor of recurrence or survival in 11 studies and was included as a predictor in these models. Six models had a dichotomous measure of tumor morphology [22,23,24,27,28], three models incorporated a continuous measure of tumor morphology [25,29,30] and two models used cut-offs of composite measures of tumor morphology (Tumor Burden Score and Modified Tumor Burden Score) [21,26]. The Tumor Biology Score did not include a direct measure of tumor morphology but incorporated the CRS as a predictor, in which tumor morphology is two of the five variables [20].

No other single clinical or pathological factor consistently predicted recurrence or survival in more than one study. Demographic factors (age and sex) were modest predictors of overall survival and were only included in the Pawlik–Paredes clinical score and the prediction model for 10-year overall survival by Buisman et al., 2022 [25,30]. Regional lymph node involvement was the most common prognostic factor from the primary tumor and was included in nine models [19,21,22,23,24,25,26,27,30]. The carcinoembryonic antigen (CEA) level was evaluated in 11 models but only included in four [21,25,26,30]. Carbohydrate antigen 19-9 (CA 19-9) was included in two models using different cut-off values [26,28]. The significance of resection margin status was inconsistent in seven studies that assessed this variable; the GAME score excluded margin status because this is not available preoperatively, whereas the prediction model for 10-year overall survival by Buisman et al., 2022 included resection margin status [19,20,21,23,27,29,30]. Extrahepatic disease was evaluated in six models but only significant in three models [20,21,23,26,28,30]. Preoperative chemotherapy was a significant predictor of recurrence or survival in six models and included five models [19,20,23,25,29,30].

## 5. Evaluation of Individual Prognostic Models Incorporating *RAS* Mutation Status in CRLM

### 5.1. RAS-Informed Treatment Algorithms

Passot et al. from the MD Anderson Cancer Center analyzed the outcomes of 524 patients with *RAS* mutant CRLM undergoing curative liver resection between 2005 to 2015. Survival analysis demonstrated that patients with *RAS* mutation had a statistically significant shorter median overall survival (50.9 months) than patients with wild-type *RAS* (72.6 months). However, the median overall survival for patients with codon 12 and codon 13 mutations was not statistically significant. Multivariable analysis of potential predictors of overall survival was only performed for patients with *RAS* mutation (n = 212), which found that three factors were independently associated with worse overall survival in this group: node-positive primary tumor (HR 2.59, 95% CI 1.11–6.05), tumor > 3 cm (HR 2.28, 95% CI 1.37–3.81) and >7 cycles of preoperative therapy (HR 1.9, 95% CI 1.15–3.12). The hazard ratio of *RAS* mutation status was not determined in this study, limiting the ability to measure the effect of *RAS* mutation on survival. The median survival of patients with *RAS* mutation and three risk factors was similar to patients treated with chemotherapy alone. In addition, there were no four-year survivors with all three risk factors. The authors concluded that curative resection for these high-risk may be “ill-advised”. Instead, they proposed a treatment schema in which high-risk patients are considered for further systemic therapy or alternatives such as hepatic arterial infusion therapy to reduce recurrence [19].

### 5.2. Genetic and Morphologic Evaluation Score

Margonis et al. developed the Genetic and Morphological Evaluation (GAME) score based on data from a development cohort of 506 patients from the Johns Hopkins Hospital and a validation cohort of 747 patients from the Memorial Sloan Kettering Cancer Center that underwent complete resection of CRLM between 2000 to 2015. In addition to being one of the first models to include *KRAS* mutation status as a surrogate of tumor biology, the GAME score differentiates itself from the previous models by incorporating the tumor burden score (TBS), a redefined CEA level (20 ng/mL) and extrahepatic disease [21]. The tumor burden score (TBS) is a continuous variable and composite measure of tumor morphology and has been previously described. The TBS is divided into three prognostic zones (TBS < 3, TBS 3 to <9, and TBS ≥9) that correspond to increasing tumor burden and captures more prognostic information than dichotomizing tumor size or the maximum number of tumors [34]. The optimal CEA level was determined based on the ROC curve analysis of its development cohort. Multivariable analysis of prognostic factors in patients with resected CRLM identified six independent predictors of overall survival that can be determined preoperatively: *KRAS* mutated tumors (HR 1.50, 95% CI 1.13–2.00), CEA ≥ 20 (HR 1.90, 95% CI 1.43–2.47), TBS 3 to <9 (HR 1.66, 95% CI 1.14–2.44), TBS ≥ 9 (HR 3.23, 95% CI 2.01–5.07), primary tumor lymph node metastasis (HR 1.55, 95% CI 1.14–2.10) and extrahepatic disease (HR 2.10, 95% CI 1.35–3.22). These independent predictors were combined into a simple, seven-point weighted score: *KRAS* mutated tumors (1 point), CEA level of ≥20 mg/mL (1 point), TBS < 9 (1 point), TBS ≥9 (2 points), primary tumor lymph node metastasis (1 point) and extrahepatic disease (2 points). An incremental GAME score was associated with worse overall survival—patients with 0 points had an estimated five-year overall survival of 81.5% compared to 0% five-year overall survival in patients with ≥6 points. The discrimination of the GAME score (C-index of 0.645 and AIC of 2219) outperformed the CRS (C-index of 0.578 and AIC of 2266). Subsequent external validation by international, multi-institutional cohorts has demonstrated strong calibration of the GAME score and higher discrimination than the CRS and m-CS [35,36]. The GAME score has also been used as a part of a risk stratification framework in observational studies to assess treatment strategies in resectable CRLM [37].

### 5.3. Modified Clinical Score

Brudvik et al. developed the modified clinical score (m-CS) from a cohort of 564 patients from the MD Anderson Cancer Center between 2005 to 2013 following an investigation of the impact of *RAS* mutation on the CRS. Node-positive primary tumor (HR 2.07, 95% CI 1.361–3.165), largest liver metastasis >50 mm in diameter (HR 1.636, 95% CI 1.180–2.905), and *RAS* mutation (HR 2.693, 95% CI 1.922–3.772) were identified as independent predictors for overall survival in the multivariable analysis and incorporated in a simple 3-point clinical score. The disease-free interval of <12 months and the number of CRLM and CEA levels were nonsignificant even with optimal cut-off values identified by receiver operating characteristic (ROC) and AUC analysis. Kaplan-Meier survival curves for overall survival and recurrence-free survival in the model development and international multicentre validation cohorts showed a decrease in survival with the addition of each point in the m-CS, which also stratified these outcomes better than the CRS. Discrimination of the m-CS (C-index 0.69) outperformed the CRS (C-index 0.57) for overall survival in the development cohort, but calibration was not reported. The proportion of patients with CRLM >50 mm was relatively low (11.2%) in the model development cohort, and the optimal cut-off was not determined as with other continuous variables. This raises whether 50 mm is the optimal size cut-off as a predictor in contemporary cohorts. Nevertheless, this study found that the addition of *RAS* mutation status could create a m-CS that would outperform the CRS [22].

### 5.4. Tumor Biology Score

The Tumor Biology Score proposed by Wang et al. is based on an analysis of 300 patients with CRLM from 2006 to 2016 treated with neoadjuvant or conversion chemotherapy before liver resection. Poor preoperative response to chemotherapy was defined as progressive disease or a decrease in the diameter of target lesions by less than 30%. There was wide variation in the amount of preoperative chemotherapy delivered, with patients receiving between one and 16 cycles. Due to the selection criteria, this cohort was characterized by a higher proportion of patients with high-risk features such as *KRAS* mutation (63.3%), CRS > 2 (51.6%), poor response to preoperative chemotherapy (60.7%), synchronous CRLM (88.3%) and extrahepatic disease (18.0%). Multivariate analysis identified three independent prognostic factors for overall survival: CRS > 2 (HR 4.245, 95% CI 1.758–8.260), *KRAS* mutation (HR 2.196, 95% CI 1.372–3.515), and poor response to preoperative chemotherapy (HR 2.054, 95% CI 1.025–4.119). Morphological factors in original CRS (largest hepatic tumor >5 cm and number of hepatic tumors >1) were not statistically significant on multivariate analysis in this cohort. A simple three-point risk score was developed by assigning one point for each predictor regardless of the hazard ratio. The 5-year overall survival for patients with 0, 1, 2 and 3 points were 63.7%, 49.6%, 33.3% and 14.1%, respectively. Discrimination of the Tumor Biology Score (C-statistic 0.642, 95% CI 0.570–0.713) was higher compared to the CRS (0.585; 95% CI 0.520–0.549) and m-CS (0.615; 95% CI 0.531–0.699) [20].

### 5.5. Extended Clinical Score

The e-CS improves on the m-CS by incorporating molecular biomarkers from multigene panel testing. The study by Lang et al., 2019 performed molecular profiling on 139 tissue samples randomly selected out of 822 patients who had undergone resection of CRLM between 2008 and 2018. Next-generation sequencing of 720 cancer-related genes was correlated with clinicopathological and outcome data from a prospectively maintained database to identify prognostic biomarkers. Alterations in the *RAS/RAF* pathway, *SMAD* family, *PIK3CA*, and *TP53* were negative predictors for overall survival. For simplicity, only alterations in the *RAS-RAF* pathway and *SMAD* family, which had the highest prognostic significance for oncologic outcomes, were included in the e-CS. The e-CS modifies the m-CS by replacing *RAS* mutation with alterations in the *RAS-RAF* pathway and adding alterations in the *SMAD* family as a predictor. Compared to the m-CS, the four-point e-CS improved the stratification of patients according to overall survival in this cohort. The high concordance of *RAS/RAF* and *SMAD* alterations between primary CRC and CRLM means that the e-CS can be applied preoperatively [24,38].

The findings in this study are supported by recent studies, which show that combined somatic mutations such as *RAS*, *TP53*, and *SMAD4* are associated with a worse prognosis than mutations in *RAS* alone [39,40]. However, as alluded to by the authors, the cost of extended molecular profiling is a limiting factor in achieving an adequate sample size and statistical power [24]. As next-generation sequencing technology continues to develop and costs continue to decline, the conditions to develop and validate models such as the e-CS are not unforeseeable.

### 5.6. Paredes-Pawlik Clinical Score

The Paredes-Pawlik Clinical Score is the first prediction model to apply a machine learning approach to predict recurrence after resection of CRLM. The full model was developed from data of an international, multi-institutional cohort of 1406 patients treated between 2001 and 2018. Implementation of the prediction model is facilitated by an easy-to-use online calculator (https://paredespawlikcalc.shinyapps.io/CRLM/, accessed on 1 June 2022) that incorporates 11 prognostic variables: age, sex, primary tumor location, American Joint Committee on Cancer (AJCC) T stage, primary tumor lymph node metastasis, number of CRLM (>1), size of the largest tumor (>5 cm), CEA level >200 ng/mL, *KRAS* status (unknown, wild-type or mutated), disease-free interval <12 months and preoperative chemotherapy. The clinical score demonstrates good discriminative ability to predict recurrence: 1-year recurrence (AUC 0.693, 95% CI 0.684–0.704), 3-year recurrence (AUC 0.669, 95% CI 0.661–0.677) and 5-year recurrence (AUC 0.669, 95% CI 0.661–0.679). In addition, the Paredes-Pawlik score outperformed the CRS (AUC 0.527, 95% CI 0.514–0.538) and m-CS (0.525, 95% CI 0.514–0.533) in predicting 1-year recurrence. Calibration of the Paredes-Pawlik Clinical Score demonstrated good model accuracy. A useful feature of this model is its ability to include risk prediction for patients who may not have known *RAS* mutation status before liver resection [25].

### 5.7. Nomograms for Predicting Recurrence after Resection of CRLM

Liu et al. have published two nomograms (2019 and 2021) for predicting recurrence after resection of CRLM in different cohorts [23,27]. The development cohort in the first study included 447 patients from the Peking University Cancer Hospital treated with preoperative chemotherapy followed by resection of CRLM from 2010 to 2017. Multivariate analysis identified five independent factors for disease-free survival: *RAS* mutation (HR 1.316, 95% CI 1.030–1.682), node-positive primary (HR 1.341, 95% CI 1.033–1.788), CRLM larger than 5 cm (HR 1.517, 95% CI 1.062–2.168), multiple CRLM (>1), primary tumor size and tumor progression on preoperative chemotherapy (HR 1.415, 95% CI 1.033–1.939). A specific score was assigned to each factor to develop a point-based prognostic nomogram to predict disease-free survival: *RAS* mutation: 5 points, node-positive primary: 6 points, CRLM larger than 5 cm: 8 points, multiple CRLM (>1): 10 points and tumor progression on chemotherapy: 5 points. The C-statistic for this 34-point nomogram was 0.675 and externally validated in an independent cohort of 117 patients from the Sun Yat-Sen University Oncology Hospital and Harbin Medical University Cancer Hospital between 2013 and 2017, demonstrating a C-statistic of 0.77 [23].

The second study by Liu et al., 2021 developed a nomogram to predict progression-free survival after resection of CRLM. This study shares many similarities to their earlier work, the main distinguishing feature being the inclusion of patients who had not received neoadjuvant chemotherapy, which made up 39.0% of this cohort. This larger cohort appeared to overlap with the former study with 469 patients at the Peking University Cancer Hospital between 2008 and 2018 in the development cohort and 145 patients from the Sun Yat-Sen University Oncology Hospital between 2009 and 2018 in the validation cohort. In addition, researchers included two smaller cohorts from the Changhai Hospital (n = 92) and Fudan University Shanghai Cancer Center (n = 63) in the development and validation cohort. Multivariable analysis identified five independent factors, four of which were included in the first nomogram: *RAS* mutation (HR1.791, 95% CI 1.407–2.281), node-positive tumor (HR 1.961, 95% CI 1.520–2.530), CRLM larger than 5 cm (HR 2.574, 95% CI 1.855–3.571) and multiple CRLM (>1) (HR 2.368, 95% CI 1786–3.140). The fifth independent factor in this study was primary tumor location (HR 1.733, 95% CI 1.311–2.291) rather than tumor progression on preoperative chemotherapy. Based on these results, the authors developed a 43-point nomogram with a C-statistic of 0.682. The authors’ earlier work was not referenced in this second study, and the context in which these relatively similar nomograms are applicable is unclear [23,27].

### 5.8. Comprehensive Evaluation of Relapse Risk Score

The Comprehensive Evaluation of Relapse Risk (CERR) score is a five-point prognostic score to predict relapse-free survival after curative-intent resection of CRLM. The study aimed to develop a prognostic score that improved the GAME score by considering the distribution of CRLM (i.e., unilobar or bilobar) in the TBS, *NRAS*, and *BRAF* mutation status and CA19-9. The authors introduced the modified tumor burden score (mTBS) and the use of a natural constant, √e, based on the approximation of this constant (1.6487) to the hazard ratio of bilobar metastasis (1.63) in this cohort; the TBS is multiplied by √e in patients with bilobar metastasis. Similar to the TBS, mTBS was divided into three intervals: <5, 5–12, and ≥12). *KRAS*, *NRAS,* and *BRAF* were considered one predictor. The optimal cut-off point for CA-19-9 was determined at 200 U/mL using a bioinformatics tool for biomarker assessment and outcome-based cut-point optimization. A six-point prognostic model: node-positive primary (1 point), CEA >200 ng/mL or CA19-9 >200 U/mL (1 point), *KRAS*/*NRAS*/*BRAF*-mutated tumor (1 point) extrahepatic disease (1 point), mTBS between 5 and 11 (1 point) and mTBS more than 12 (2 points). The CERR (AUC 0.69 at 24 months, 95% CI 0.64–0.73) demonstrated better discrimination than the CRS (0.625) and GAME (0.636) scores in this cohort [26].

### 5.9. Modifying Effects of Perioperative Chemotherapy on Prediction Models Incorporating RAS Mutation Status

Takeda et al. hypothesized that the accuracy of clinical scores that include *RAS* status is affected by the administration of perioperative chemotherapy. Multivariable analysis of 393 patients who had undergone curative-intent resection for CRLM between 2010 and 2016 identified three prognostic factors for overall survival: *RAS* mutation (1.729, 1.172–2.552), CRLM  ≥4 (HR 1.770, 95% CI 1.199–2.612) and CA19-9  ≥100 U/mL (HR 2.092, 95% CI 1.336–3.275). The C-index was 0.65 for overall survival in the newly developed model. In addition, the authors compared the overall survival of 485 patients who had received perioperative chemotherapy and 165 patients who had undergone surgery alone and found that the newly developed model and m-CS did not stratify overall survival and the CRS in patients who had not received perioperative chemotherapy. Although the authors suggest that risk scores incorporating *RAS* may have limited value in patients who do not receive chemotherapy, this remains to be determined as the observation may have been influenced by patient selection and confounding factors [28].

### 5.10. Contour Prognostic Model

The contour prognostic model is adapted from the Metroticket model to predict overall survival based on the largest tumor diameter and number of metastases as independent continuous variables [29]. This overcomes the limitations of dichotomizing tumor morphology, such as loss of information, underestimating the extent of variation in outcome between groups, and concealing non-linearity between the variable and outcome [41]. Multivariable analysis identified six factors that were significantly associated with overall survival: largest diameter of CRLM (continuous variable, HR 1.11, 95% CI 1.06–1.16), number of CRLM (continuous variable, HR 1.06, 95% CI 1.03–1.09), *RAS* mutation status (HR 1.76, 95% CI 1.42–2.18), age (continuous variable, HR 1.02, 95% CI 1.01–1.03), primary lymph node metastasis (HR 1.58, 95% CI 1.23–2.03) and prehepatectomy chemotherapy (HR 1.47, 95% CI 1.04–2.08). Primary lymph node metastasis was not included because this information may not be available for patients undergoing a liver-first approach or simultaneous resection of the primary CRC and CRLM in synchronous disease. Prehepatectomy chemotherapy was also excluded because of heterogeneity in its use between institutions. Furthermore, age, primary lymph node status, and prehepatectomy chemotherapy only resulted in limited improvement in model discrimination. The contour plots of predicted 5-year overall survival probability according to the largest tumor diameter, number of metastases, and *RAS* mutation status had a C-statistic of 0.625 for mutated *RAS* and 0.657 for wild-type *RAS* [29].

### 5.11. Predicting 10-Year Overall Survival after Resection of Colorectal Liver Metastases

Buisman et al. recently developed a model for predicting 10-year overall survival from a cohort of 4539 patients. Despite the long inclusion period, 82% underwent resection after 2000. This model has the highest number of predictors with 15 independent prognostic factors. A web-based calculator (https://www.oncocalculators.com/, accessed on 1 June 2022) is available to facilitate the application of this model. The AUC of 0.73 for both cohorts included in this study is the highest discrimination among the models reviewed and outperformed the CRS (AUC 0.62) and GAME score (AUC 0.66). A simplified risk score with 13 dichotomous variables stratified patients into four prognostic groups with 10-year overall survival ranging from 12% to 57%. This is the first prediction model to include *KRAS* and *BRAF* mutation status as separate variables. Both these factors were confirmed as independent predictors for 10-year overall survival with an adjusted hazard ratio of 1.59 (95% CI 1.46–1.73) and 1.69 (1.42–2.01), respectively). Although hepatic arterial infusion pump (HAIP) therapy is a predictor, the model included more than 3000 patients who did not receive HAIP therapy and can be applied to patients who do not receive HAIP. Although other models have been designed to predict outcomes before liver resection, this model is more intended to predict survival following resection as it includes histopathological growth patterns and CRLM resection margins which are not available preoperatively [30].

## 6. Prognostic Value of *RAS* Mutation Status in Colorectal Liver Metastases

The current narrative review evaluates contemporary prognostic models that incorporate molecular biomarkers to predict survival after resection of CRLM. Anti-*EGFR* monoclonal antibodies, such as cetuximab and panitumumab, have established efficacy in advanced CRC and have been used in clinical practice since 2004 [42]. *RAS* mutation testing gained importance following evidence demonstrating that wild-type *KRAS* was a condition for response to anti-*EGFR* therapy [17,43]. A meta-analysis of *KRAS* mutations and survival after resection of CRLM in 2015, which included 14 studies and 1809 patients from 1982 to 2014, reported a *KRAS* mutation rate of approximately 28.0%. Ten studies in the referenced meta-analysis only included the most common *KRAS* codon (12 and 13) alterations [18]. The higher *RAS* mutation rate in the studies included in this review likely reflects the implementation of extended *RAS* mutational analysis for patients considered for anti-*EGFR* therapy (*KRAS* and *NRAS* isoforms; codons 12 and 13 of exon 2; codons 59 and 61 of exon 3; and codons 117 and 146 of exon 4) [44]. Specific *KRAS* mutations are associated with different biologic characteristics and may have prognostic implications as suggested by variation in survival outcomes after resection in CRLM [45]. For example, patients with *KRAS* G12V or G12S mutation have been reported to have worse overall survival compared to patients with a *KRAS* codon 13 mutations [46]. In contrast, patients with codon 13 mutations have a higher risk of overall extrahepatic recurrence and lung-specific recurrence than patients with codon 12 mutations [47]. 

Collaboration between institutions to develop and validate prognostic models in resectable CRLM has enabled the impact of *RAS* mutation to be evaluated in large cohorts. Furthermore, these studies largely include patients after introducing irinotecan and oxaliplatin as components of combined cytotoxic chemotherapy and anti-*EGFR* therapy in the early 2000s [4]. The 12 prognostic models that have included *RAS* mutation status in multivariable analysis confirm that *RAS* mutation status is an independent prognostic factor for overall survival (eight studies) and recurrence-free survival (four studies) in the era of modern chemotherapy. 

Despite its role as a negative prognostic factor in CRLM, incorporating *RAS* mutation status into risk scores has only contributed to modest improvements in predictive performance. Nine of the 11 prognostic models reported a hazard ratio of less than 2.00 for *RAS* mutation status. In contrast, one model (e-CS) did not find a statistically significant *KRAS* or *NRAS* alone effect on overall survival. These findings are consistent with an updated systematic review and meta-analysis on RAS mutation status in patients with CRLM by Pikoulis et al., 2021 concluded that the effect of *RAS* mutation on overall survival and recurrence-free survival had been previously overestimated. The hazard ratios for overall survival and recurrence-free survival in this updated study were 1.49 (95% CI 1.30–1.71) and 1.36 (95% CI 1.22–1.51), respectively [48]. Comparison to the meta-analysis by Brudvik et al., 2015 which established the prognostic value of *KRAS* and reported corresponding hazard ratios of 2.24 (95% CI 1.76 to 2.85) and 1.89 (95% CI 1.54–2.32) suggests that the effects of *RAS* mutation status are lower than previously described [18].

Several reasons can explain the reduced prognostic value of *RAS* status. First, studies that have included *RAS* status as part of a prognostic model have a larger sample size when compared to studies that have evaluated *RAS* mutation status as a single prognostic factor [18]. Consequently, a higher number of events (recurrence or death) allows a higher number of prognostic factors to be adjusted in the multivariable analysis and a more precise measure of the effects of *RAS* status. Second, a more contemporary cohort of patients (after 2000) is included in these studies [4]. Advancements in systemic therapy agents, treatment approaches and molecular profiling in CRLM have led to better patient selection, improved survival and reduced the impact of *RAS* mutation. Lastly, using *RAS* status as a dichotomous variable may lead to a loss of information on the variability in patients with wild-type and mutated *RAS* [41]. The prognostic value of specific *RAS* mutations and their interaction with other molecular markers is a key issue in current prognostic research. Evaluating the impact of these molecular alterations on the performance of prognostic models and updating existing prognostic models to reflect advancements in our understanding of these molecular alterations are areas for ongoing research. 

## 7. Conclusions

This review has provided an overview of prediction models incorporating *RAS* mutation status to estimate survival probabilities after resection of CRLM. These models offer robust evidence that *RAS* mutation status is a consistent and independent predictor of survival across different populations. However, the predictive performance of models incorporating *RAS* mutation status remains modest, which emphasizes the need to combine *RAS* mutation status with established and novel predictors to develop prognostic models that provide better survival estimates. The CRS continues to be a reference point for evaluating the predictive performance of novel prediction models, but the GAME score and m-CS are emerging as new standards of comparison. Risk stratification using these newer models continues to demonstrate prognostically distinct groups in patients with technically resectable CRLM. Although the expanding resectability criteria partly explain this, the influence of tumor biology is incompletely understood. Incorporating *RAS* mutation status in clinical risk scores has shown how the effects of tumor biology and established clinicopathological factors can be adjusted and assessed simultaneously. This has motivated prognostic models to explore the impact of other cancer-based molecular biomarkers beyond *RAS*, which will be the next frontier in clinical prediction models to inform clinical decision-making in CRLM.

## Figures and Tables

**Table 1:** Summary of prognostic models incorporating *RAS* mutation status.

First Author, Year, Reference	Institution	Inclusion Period	Inclusion Criteria	Patients (*n*) by Cohort		Predicted Outcome	Model, Number of Predictors and Maximum Score
				Development cohort (DC)	Validation cohort (VC)		
Fong 1999 [11]	MSKCC, USA	1985–1998	Consecutive patients after complete resection of CRLM	1001	NA	Overall survival	Clinical Risk Score (CRS) 5 predictors 5 points
Passot 2017 [19]	MD Anderson Cancer Center, USA	2005–2015	Known *RAS* mutation status	524	NA	Overall survival	Risk score for *RAS* mutated tumors 3 predictors 3 points
Wang 2017 [20]	Peking University Cancer Hospital, China	2006–2016	Known *RAS* mutation status and preoperative chemotherapy	300	NA	Overall survival	Tumor Biology Score 3 predictors 3 points
Margonis 2018 [21]	DC: JHH, USA VC: MSKCC, USA	2000–2015	Known *RAS* mutation status	502	747	Overall survival	Genetic and Morphology Evaluation (GAME) score 5 predictors Weighted score, 7 points
Brudvik 2019 [22]	DC: MD Anderson Cancer Center, USA VC: International multicentre cohort	2005–2013	Known *RAS* mutation status	564	608	Overall survival	Modified Clinical Score (m-CS) 3 predictors 3 points
Liu 2019 [23]	DC: Peking University Cancer Center, China VC: Sun Yat-Sen University Oncology Hospital, Harbin Medical University Cancer Hospital, China	2010–2017	Preoperative chemotherapy and resection for CRLM	447	117	Disease-free survival	Nomogram 5 predictors 0–34 points
Lang 2019 [24]	Universitätsmedizin Mainz, Germany	2008–2018	139 randomly selected patients out of 822 patients from a prospective database	139	NA	Overall survival	Extended Clinical Risk Score (e-CS) 4 predictors 4 points
Paredes 2020 [25]	International multi-institutional database	2001–2018	Resection of CRLM, with known and unknown *RAS* mutation status. Machine learning approach	703	703	Recurrence-free survival	Paredes-Pawlik Score calculator 11 predictors Online calculator (https://paredespawlikcalc.shinyapps.io/CRLM/, accessed on 1 June 2022)
Chen 2020 [26]	Zhingshan Hospital, China	2010–2018 in DC, 2018 only in VC	Patients with available data on *KRAS/NRAS/BRAF* mutation status	787	162	Relapse-free survival	Comprehensive Evaluation of Relapse Risk (CERR) score 5 predictors Weighted score, 6 points
Liu 2021 [27]	DC: Peking University Cancer Hospital, Fudan University Shanghai Cancer Center, China VC: Sun Yat-Sen University Cancer Hospital, Changhai Hospital, China	2008–2018	Patients who underwent curative-intent resection of CRLM	532	237	Progression-free survival	Nomogram Five predictors 0–43 points
Takeda 2021 [28]	DC: Cancer Institute Hospital, Japan. VC: Multicentre cohort, Japan	2010–2016	Patients who underwent curative-intent resection of CRLM	341	309	Overall survival	Risk score 3 predictors 0–3 points
Kawaguchi, 2021 [29]	DC: MD Anderson Cancer Center, USA VC: International multi-institutional cohort	1998–2017	Known *RAS* mutation status	810	673	Overall survival	Contour prognostic model and Excel 5-year OS calculator based on *RAS* mutation status and diameter and number of lesions as continuous variables
Buisman 2022 [30]	DC: MSKCC, USA VC: Erasmus MC, Netherlands	1992–2019	Consecutive patients after complete resection of CRLM	3064	1048	Overall survival	Complete model: 15 predictors Online calculator (calculator www.oncocalculators.com, accessed on 1 June 2022) Simplified risk score: 13 dichotomized predictors −3 to 17 points

**Table 2:** Summary of the performance of prognostic models incorporating *RAS* mutation status to predict outcomes post CRLM resection.

Author, Year, Reference	Model Discrimination Concordance Statistic (95.0% Confidence Interval)			Model Calibration		Prognosis		
	Development Cohort	Validation Cohort	Comparison to Other Prediction Models	Calibration Method	Stated Interpretation	Risk Groups	Score	Survival
Fong 1999 [11]	NR	NR	NR	NR	NR			5-year OS (%)
						0 (n = 52)	0	60
						1 (n = 262)	1	44
						2 (n = 350)	2	40
						3 (n = 243)	3	20
						4 (n = 80)	4	25
						5 (n = 14)	5	14
Passot 2017 [19]	NR	NR	NR	NR	NR	RAS mutated		Median OS (months)
						0 (n = 23)	0	58
						1 (n = 96)	1	57
						2 (n = 51)	2	41
						3 (n = 14)	3	21.5
Wang 2017 [20]	0.642 (0.570–0.713)	NR		NR	NR			5-year OS (%)
			CRS 0.585			0 (n = 70)	0	63.7
			(0.474–0.696)			1 (n = 121)	1	49.6
			m-CR 0.615			2 (n = 75)	2	33.3
			(0.531–0.699)			3 (n = 34)	3	14.1
Margonis 2018 [21]	GAME: C-statistic 0.645 (0.598–0.692) AIC 2219	International cohort 0.61 ^†^	CRS: C-statistic 0.578 (0.530–0.625) AIC 2266	NR in model development. In a subsequent external validation (Sasaki 2021), researchers assessed calibration curves for each score by comparing the probability of observed and predicted mortality with ordinary least squares regression [34].	Correlation and calibration coefficients for linear regressions of observed vs predicted mortality of GAME were R^2^ = 0.98 and 1.13 at one 2 years, R^2^ = 0.98 and 1.00 at 5 years after hepatic resection.	(n = DC, VC) Low (n = 121, 171) Medium (n = 310, 402) High (n = 71, 174)	0–1 2–3 4–7	5-year OS (%) JHH, MSKCC 73.4, 76.2 50.6, 63.7 11.3, 36.5
Brudvik 2019 [22]	C-statistic 0.69 (0.62–0.76)		CRS: C-statistic 0.57 (048–0.65)	NR	NR	0 (n = 88) 1 (n = 277) 2 (n = 185) 3 (n = 14)	0 1 2 3	Median OS (months) 15 Kaplan-Meier curves demonstrated a statistically significant difference between patients with m-CS scores of 0 and 1, 1 and 2, and 2 and 3.
Liu 2019 [23]	0.675	0.77	NA	Calibration curves with bootstrapped samples.	A calibration plot for the probability of survival at 1, 3, and 5 years demonstrated good calibration between the prediction by the nomogram and the actual observation.	Quartile 1 Quartile 2 Quartile 3	0–10 11–23 23–34	Median DFS (months) 17 8 3
Lang 2019 [24]	NR	NR	NR	NR	NR			Median OS ‡
								(days, months)
						Score 1 (n = 123)	1	1695, 60.5
						Score 2 (n = 43)	2	1183, 42.3
						Score 3 (n = 22)	3	631, 22.5
						Score 4 (n = 5)	4	368, 13.1
Paredes 2020 [25]	1-year recurrence 0.693 (0.684–0.704) 3-year recurrence 0.669 (0.661–0.677) 5-year recurrence 0.669 (0.661–0.679)	Similar model performance	CRS: 1-year recurrence 0.527 (0.514–0.538) m-CR 1-year recurrence 0.525 (0.514–0.533) Researchers noted similar trends for 3- and 5-year recurrence.	Calibration curves of the alternative score with and without adjustment for *KRAS* status among individuals with known *KRAS* status in the 100 imputed model design and validation cohorts.	Calibration curves for the model design and validation demonstrated good model accuracy.	Low Medium High	Lower quartile Medium two quartiles Upper quartile	Increase of 0.25 in the alternative score was associated with a 61% increase in recurrence (HR, 1.61, 95.0% CI 1.40–1.85) and a 39.0% increased risk of death (HR, 1.39; 95.0% CI 1.18–1.63)
Chen 2020 [26]	0.690 (0.650–0.730)	0.630 (0.605–0.655)	CRS 0.586 (0.560–0.612) GAME score 0.602 (0.575–0.629)	Calibration curves with bootstrapped samples.	At a probability between 0 and 0.23, the CERR score model may slightly overestimate the RFS risk; when the probability is higher than 0.23, the model may slightly underestimate the probability. The CERR score model showed a good fit and calibration with the ideal curve.	(n = DC, VC) Low (n = 118, 37) Medium (n = 454, 94) High (n = 105, 31)	0–1 2–3 4–6	Median OS (months) 23.7 12.7 7.3
Liu 2021 [27]	0.696	0.682	0.642	Calibration curves with bootstrapped samples.	A calibration plot for the probability of survival at 1, 3, and 5 years demonstrated good calibration between the prediction by the nomogram and the actual observation.	Low (n = 344) High (n = 425)	0–16 17–43	Progression-free survival (%) 30 months 10 months
Takeda 2021 [28]	0.65	NR	Comparison to CRS and m-CS performed but C-statistic not reported.	NR	NR	0 (n = 94) 1 (n = 163) 2 (n = 68) 3 (n = 16)	0 1 2 3	Visual assessment of Kaplan-Meier survival curves demonstrates a difference in overall survival between different scores. OS by risk score NR.
Kawaguchi 2021 [29]	Mutated *RAS* 0.629 (s.e. 0.021) Wild-type *RAS* 0.625 (s.e. 0.022)	Mutated *RAS* 0.644 (s.e. 0.026) Wild-type *RAS* 0.624 (s.e. 0.026)	CRS: 0.563 GAME: 0.606	Comparing the average overall survival probability predicted by the prognostic model with the overall survival probability estimated by the Kaplan-Meier method after grouping predicted survival by quintile.	Observed survival lay within a 10% margin of error around predicted survival for both mutant *RAS* and wild-type *RAS* disease.	Contour plots and Excel^®^ 5-year OS calculator for mutated and wild-type *RAS* tumors	Largest diameter and number of CRLM as continuous variables	5-year OS (%) Example: 3 CRLM and largest CRLM 5 cm *RAS* wild-type: 43.0 *RAS* mutated: 49.5
Buisman 2022 [30]	0.73 (0.70–0.75)	0.73 (0.68–0.78)	CRS 0.62 (0.59–0.64) GAME 0.66 (0.64–0.69)	Assessed visually by plotting the predicted probability against the actual observed frequency of predicted outcomes at 10 years and using cross-validation.	Calibration plots showed a slight overestimation of the model developed in Erasmus MC. Calibration was good in the model developed in MSKCC and validated in Erasmus MC.	1 (n = 692) 2 (n = 993) 3 (n = 1483) 4 (n = 944)	Simplified risk score ≤3 4–5 5–8 9–13	10-year OS (%) 57% 38% 24% 12%

AIC—Akaike Information Criterion; DC—development cohort; VC—validation cohort; NR—not reported; OS—overall survival. ^†^ Median OS reported in days. and approximated in months by dividing the number of days by 28.

**Table 3:** *RAS* analysis in included studies.

Study, Year, Reference	Patients with Known *RAS* Mutation Status, n	*RAS* Mutation Rate, n (%)	*RAS* Hazard Ratio	*RAS* 95.0% CI	*RAS* Isoforms: Codons Tested
Passot 2017 [19]	524	212 (40.5)	NR	NR	*KRAS*: 12, 13, 61, 146 *NRAS*: 12, 13, 61
Wang 2017 [20]	300	190 (63.3)	2.20	1.37–3.52	NR
Margonis 2018 [21]	1249	466 (37.3)	1.50	1.13–2.00	*KRAS*: 12, 13, 61
Brudvik 2019 [22]	564	205 (36.3)	2.69	1.92–3.77	*KRAS*: 12, 13, 61, 146 *NRAS*: 12, 13, 61
Liu 2019 [23]	564	227 (46.2)	1.32	1.03–1.68	NR
Lang 2019 [24]	139	38 (37.9)	1.44	0.90–2.33	NR RAS analysis was included in the assessment of 720 genes catalogued in the cancer gene census.
Paredes 2020 [25]	707	268 (37.9)	NR	NR	*KRAS*: 12, 13, 61, 117, 146 *NRAS*: 12, 13, 61, 146
Chen 2020 [26]	949	408 (43.0)	1.79	1.32–1.90	*KRAS*: 12, 13, 61, 117, 146 *NRAS*: 12, 13, 61, 146
Liu 2021 [27]	769	200 (37.6)	1.73	1.41–2.28	NR
Takeda 2021 [28]	341	145 (42.5)	1.73	1.17–2.55	*KRAS*: 12, 13, 59, 61, 117, 146 *NRAS*: 12, 13, 59, 61, 117, 146
Kawaguchi 2021 [29]	810	364 (44.9)	1.76	1.42–2.18	*KRAS*: 12, 13, 61, 146 *NRAS*: 12, 13, 61
Buisman 2022 [30]	1567	639 (41.0)	1.58	1.46–1.73	NR

**Table 4:** Predictors considered and included in prognostic models incorporating RAS to predict outcomes after resection of CRLM.

	Univariable—Evaluated	Multivariable—Evaluated	Multivariable—Significant	Multivariable—Included in Model	Demo- Graphic Factors		Primary Tumour Factors			Tumour Markers		Metastatic Disease Factors												Treatment Factors			Molecular Factors		
					Age	Sex	Primary Tumour Location	Primary Tumour—T Stage	Primary lymph Node Involvement	Preoperative Serum CEA	Preoperative Serum CA19-9	Disease-Free Interval/Timing of CRLM	Diameter of Largest CRLM	Number of CRLM	Tumour Burden Score (TBS)	Modified TBS	Bilobar or Unilobar CRLM	Histopathological Growth Pattern	CRLM Resection Margin (R0, > 1 mm)	Pathologic Response	Extrahepatic Disease	Clinical Risk Score (CRS)	Extent of Liver Resection (Major/Minor)	Operative Blood Loss/Major Complications	Pre- or Perioperative Chemotherapy	Ablation	*RAS* Mutation	*BRAF* Mutation	*SMAD* Mutation
Fong 1999 (reference)	13	8	7	5																									
Passot 2017 [19]	18	5	3	3																									
Wang 2017 [20]	19	5	3	3																									
Margonis 2018 [21]	12	6	6	5																									
Brudvik 2019 [22]	6	3	3	3																									
Liu 2019 [23]	26	26	5	5																									
Lang 2019 [24]	NR	NR	NR	4																									
Paredes 2020 [25]	NR	NR	11	11																									
Chen 2020 [26]	11	9	5	5																									
Liu 2021 [27]	19	10	5	5																									
Takeda 2021 [28]	17	10	3	3																									
Kawaguchi 2021 [29]	12	6	6	3																									
Buisman 2022 [30]	NR	NR	15	15																									

## Data Availability

Not applicable.
